# Early-life gut bacterial community structure predicts disease risk and athletic performance in horses bred for racing

**DOI:** 10.1038/s41598-024-64657-6

**Published:** 2024-08-07

**Authors:** J. Leng, C. Moller-Levet, R. I. Mansergh, R. O’Flaherty, R. Cooke, P. Sells, C. Pinkham, O. Pynn, C. Smith, Z. Wise, R. Ellis, A. Couto Alves, R. La Ragione, C. Proudman

**Affiliations:** 1 School of Veterinary Medicine, Faculty of Health and Medical Sciences, University of Surrey, Daphne Jackson Road, Guildford, GU2 7AL UK; 2 Institute of Integrative Biology, University of Liverpool, Liverpool, L69 7ZB UK; 3 School of Biosciences, Faculty of Health and Medical Sciences, Edward Jenner Building, University of Surrey, Guildford, GU2 7XH UK; 4 Department of Earth Sciences, University College London, Gower Street, London, WC1E 6BT UK; 5 Avonvale Vets, 6 Broxell Close, Warwick, CV34 5QF UK; 6 Chasemore Farm, Orbital Veterinary Services, Bookham Road, Downside, Cobham, KT11 3JT UK; 7 Pinkham Equine Veterinary Services, Home Farm Offices, Netherhapton, Salisbury, SP2 8PJ UK; 8 Rossdales Equine Practice, Beaufort Cottage Stables, High Street, Newmarket, CB8 8JS UK; 9 Newmarket Equine Hospital, Cambridge Road, Newmarket, CB8 OFG UK; 10 Surveillance and Laboratory Services Department, Animal and Plant Health Agency, Weybridge, Addlestone, KT15 3NB UK

**Keywords:** Equine, Gut microbiota, Early-life, Respiratory disease, Athletic performance, Microbiology, Gastroenterology

## Abstract

Gut bacterial communities have a profound influence on the health of humans and animals. Early-life gut microbial community structure influences the development of immunological competence and susceptibility to disease. For the Thoroughbred racehorse, the significance of early-life microbial colonisation events on subsequent health and athletic performance is unknown. Here we present data from a three-year cohort study of horses bred for racing designed to explore interactions between early-life gut bacterial community structure, health events in later life and athletic performance on the racetrack. Our data show that gut bacterial community structure in the first months of life predicts the risk of specific diseases and athletic performance up to three years old. Foals with lower faecal bacterial diversity at one month old had a significantly increased risk of respiratory disease in later life which was also associated with higher relative abundance of faecal *Pseudomonadaceae*. Surprisingly, athletic performance up to three years old, measured by three different metrics, was positively associated with higher faecal bacterial diversity at one month old and with the relative abundance of specific bacterial families. We also present data on the impact of antibiotic exposure of foals during the first month of life. This resulted in significantly lower faecal bacterial diversity at 28 days old, a significantly increased risk of respiratory disease in later life and a significant reduction in average prize money earnings, a proxy for athletic performance. Our study reveals associations between early-life bacterial community profiles and health events in later life and it provides evidence of the detrimental impact of antimicrobial treatment in the first month of life on health and performance outcomes in later life. For the first time, this study demonstrates a relationship between early-life gut bacterial communities and subsequent athletic performance that has implications for athletes of all species including humans.

## Introduction

Gut bacterial communities have a profound influence on the health of humans and animals^1,2^ and there is a growing body of evidence to support the critical role of early-life gut microbial communities in the development of immunological competence and susceptibility to disease^2,3^. Developments in sequencing technology have enabled a better understanding of gut bacterial communities in the horse as they have in humans. Studies have described faecal bacterial communities^3–6^, mapped them to different compartments of the equine hind-gut^7^, and have characterised changes associated with intestinal disease^8–10^. Early-life colonisation of the foal gut microbiota has been described^11–13^ but its influence on health and performance in later life is unknown.

The racehorse industry is the second largest rural industry in the UK (after agriculture) when measured by economic value and employment, generating over £4 billion annually^14^. Minimising the risk of disease and injury is important for the welfare of horses used for racing; maximising the potential for athletic performance is key to successful racehorse breeding and training businesses.

The relationship between gut bacterial communities and athletic performance is unclear. Studies in mice, rats, dogs and horses^15,16^ provide evidence for changes in gut bacterial community composition in response to exercise. There is less evidence that gut bacterial community structure is associated with performance^17^.

Here we use early-life faecal bacterial community data from a large cohort of foals bred for racing along with comprehensive health and performance data from the first three years of life to determine associations between early-life gut bacterial communities and health and athletic performance in later-life.

## Methods

### The Alborada well foal study

We undertook a longitudinal study of a cohort of 52 Thoroughbred foals bred for racing. Faecal bacterial community structure was characterised at nine timepoints during the first year of life based on 16S rRNA gene sequence data from 438 samples. For each foal, respiratory, gastrointestinal, orthopaedic and soft-tissue health events were tracked from birth to three years old. For all study subjects, performance data were acquired for every participation in a regulated horserace up to three years of age.

### Study population, ethics and consent

Fifty-seven Thoroughbred foals bred for flat racing, born between the beginning of February and the end of May 2018 were recruited from five Thoroughbred stud farms in the UK (Supplementary Table 1). A priori sample size estimates for paired differences in means calculated using the pwr R package (v 1.3–0), indicated that 36 foals would provide more than 80% power to detect a medium effect size (d = 0.5) with 95% confidence. However, because of concerns about incomplete sampling and losses to follow-up over a three-year study period, we elected to over-recruit by at least 50%, resulting in a final sample size of 57 foals. Typically, foals were weaned around five months old and spent their first year on the stud farm of origin. Between nine months and one year of age horses (“yearlings”) were typically moved to other premises prior to commencement of training for racing at around two years of age. Horses from the study cohort were located in 29 different training yards with no more than three horses at the same location. Five foals (01, 06, 26, 46 and 54) were lost to follow-up before 52 weeks of age and were excluded from data analysis. Samples and data from 52 foals were carried forward for further analysis. Foals were ineligible for inclusion in the study if born by caesarean section or by assisted delivery or if the dam had received antimicrobial treatments in the three months prior to parturition. All experimental protocols were approved by the University of Surrey’s NASPA sub-committee under the project reference number NERA-2017-007-SVM. All methods were carried out in full compliance with the University of Surrey Ethics Guide and the Veterinary Surgeons Act (1966). The written informed consent of the owner or manager of each stud farm was obtained prior to commencement of the study.

### Sample collection

Spontaneously voided faecal samples were collected at nine sample points within the first year of the foals’ lives: at 2, 8, 14, 28, 60, 90, 180, 272 and 365 days old, labelled as samples points one to nine (SP01 to SP09). Faeces were stored at 4 °C and transported to the University of Surrey within 48 h. Once received, samples were aliquoted and stored at − 80 °C.

### Health and athletic performance data

Written or verbal health updates were obtained weekly for all horses reporting any veterinary investigation and/or treatment for orthopaedic, soft tissue, respiratory or gastrointestinal disease/injury. Health updates were received for an average of 127 weeks (ranging 76–185 weeks, Supplementary Table 1); observations were censored for all study participants at 156 weeks. Registered racing names were used to track race performance using a public access database (racingpost.com). Finishing position and official rating (OR), a handicap metric determined subjectively by an official of the regulatory authority (ratings increase with quality of performance), were obtained after every race start; total prize money earnings and cumulative performance metrics (total starts, total placings, total wins) were collected for all race starts until the end of December 2021. On average, 7.2 finishing positions (SD = 3.94), 4.91 ORs (SD = 3.32), and 6.57 prize money earnings (SD = 3.51) were collected per horse.

### 16S rRNA gene sequencing and data analysis

Faecal samples were thawed fully and mixed before undergoing bacterial DNA extraction. PSP spin stool kit (Stratec) was used to extract bacterial DNA using the manufacturer’s instruction, with DNA eluted in 100 µl RNA/DNA free water and stored at -20 C°. DNA was quantified using the NanoQuant plate and plate reader (both manufactured by TECAN). An aliquot of 20 µl of all extracts was sent to the Animal and Plant Health Agency (APHA, Weybridge, UK) where 16S rRNA amplicon sequencing was performed on the Miseq platform (Illumina). The V4 and V5 regions of the 16S rRNA gene were amplified using the primers U515F and U927R producing fragments 300 base pairs in length^18^. Amplification was performed using the following conditions: 95°C for 3 min, 25 cycles of 95°C for 30 s, 55°C for 35 s and 72°C for one minute, followed by 72°C for 8 min. Amplicons were purified using Ampure XP magnetic beads (Beckman Coulter). Each sample was tagged with a unique pair of indices and then sequenced using Nextera XT v2 Index kits and 2 × KALPA HiFi HotStart ReadyMix. For the second round of amplification the following PCR conditions were used: 95°C for 30 s, 55°C for 30 s, 72°C for 30 s, followed by 72°C for 5 min. The resulting amplicons were purified using Ampure XP magnetic beads. The concentration of each sample was quantified using the Quantifluor assay (Promega) and concentrations were normalised before pooling samples. Sequencing was performed on an Illumina MiSeq with 1 × 300 base reads according to the manufacturer’s instructions.

We generated a median number of sequences per sample of 96,778 (range 10,116—524,584 sequences per sample). Sequence files were transferred to the University of Surrey and uploaded along with a comma separated values file containing the metadata for the samples. Data were processed using QIIME2020.2^19^. Sequencing files were converted into a QIIME artifact (qiime tools import) and a summary of the demultiplexed reads was created (qiime demux summarize). DADA2 was used to remove lower quality bases at the ends of the reads (qiime dada2 denoise-paired)^20^. Reads were removed before base 50 for the forward reads and before 50 and after 250 for the reverse reads. The output from the quality control script was visualised (qiime metadata tabulate). Summaries of the feature table and feature data artifacts were generated (qiime feature-table). The greengenes database (March 2020 release) was downloaded and classifiers were trained on the samples (qiime feature-classifier classify-sklearn). Number of reads per bacterial classification (at phyla to family level) was generated. A table containing the number of reads for all identified bacterial families was also generated to be used for further analyses (qiime taxa collapse, qiime tools extract and biom convert).

### Data pre-processing

The biomformat R package (v 1.18.0) was used to import the QIIME feature-table.biom file and generate the raw counts matrix (683 taxa in 445 samples). Samples with less than 10,000 total counts were excluded, leaving 430 samples. The taxonomy matrix was generated using the qiime2R R package (v 0.99.6). The phyloseq R package (v 1.34.0) was used to aggregate the raw count matrix at the family level (producing a table of 217 taxa in 430 samples) and to normalise the data by rarefaction (without replacement) to the lowest sample depth (10,152 total counts). Twenty-eight taxa were removed because they were no longer present in any sample after the random subsampling. The resulting family-level, aggregated, rarefied bacterial feature count matrix contained 189 taxa in 430 samples. Alpha-diversity estimates were calculated using the pheloseq package. Seven different metrics describing alpha diversity were explored, each dealing differently with species richness and dominance. The R package phyloseqCompanion (v 0.3.1) was used to extract bacterial features from the pheloseq object and turn it into a matrix. For time-to-event and generalised linear mixed-model analyses, bacterial family counts were filtered on a per sample point basis for outlier samples and low counts. For each taxon, samples that deviate > 3 × the interquartile range of the median were removed. Taxa that had less than 3 counts in more than 20% of the samples were filtered out.

### Visualising bacterial composition of samples

Outlier samples were identified using the CLOUD method^21^. Sample 26-SP01 was the only sample identified as an outlier. NMDS plots were generated using the metaMDS function from the vegan R package (v 2.6–4). The function envfit (vegan R package) was used to fit the bacterial features onto the ordination. Bacterial features with fit statistic p < 0.001 were defined as candidates for plotting. The candidate bacterial feature with the highest goodness of fit statistic (squared correlation coefficient) is plotted first, followed by the candidate bacterial feature with the next highest squared correlation coefficient if its coordinates are at least 45 degrees apart.

### Categorisation by diversity at 28 days old

To explore potential differences associated with bacterial diversity at 28 days old (SP04) horses were grouped according to Shannon diversity metric as “high” (top quartile, n = 11), “low” (lower quartile, n = 11), and “medium” (remainder, n = 20). Difference between the groups was evaluated using one-way ANOVA (Tukey test p < 0.05). An NMDS model was built (as described previously) with the abundance of bacterial families in samples at 28 days old. Only families present in at least 10 samples were used to build the model.

### Time-to-event analysis

Cox mixed-effects models were used to analyse the length of time until the occurrence of a health event using coxme (v 2.2–16) and purrr (v 0.3.4) R packages. The data from the 16S rRNA gene sequencing (bacterial family relative abundance or alpha diversity) and a range of covariates were defined as risk factors (fixed effects) while stud farm and parity were set as random effects. Age of first diagnosis (of orthopaedic injury, soft tissue injury, gastrointestinal illness or respiratory illness) was defined as any week from the week of the sample point until 3 years of age. First events or loss of follow up happening before the sample point were set to NA. Last follow up is defined as any week from the week of the sample point until 3 years of age (156 weeks). Four different models were evaluated, with models differing in the risk factors and random effects considered. Model A included sequencing data and sex, model B included sequencing data, sex and birth weight; model C included sequencing data, sex, birth weight, mare age and gestation days; model D included sequencing data, sex, supplemental colostrum (yes/no) and age weaned. All models had stud farm of origin as a random effect and model 3 had stud farm and parity as random effects. Alpha-diversity values and bacterial family relative abundance were z-scored prior to modelling. The primary objective of using different models was not to identify the best fitting model, but rather to investigate and explore the association between metagenomic variables and time to health events, while accounting for potential confounding factors. Significant risk factors were defined as those having an HR p < 0.05. For the modelling based on bacterial family relative abundance, Benjamini and Hochberg (BH) procedure was used to correct p values for multiple testing^22^ and significant risk factors were defined as those having a BH-corrected HR with p < 0.05.

Kaplan–Meier survival analysis of horses stratified according to levels of alpha-diversity or bacterial family relative abundance was performed and log rank p-values calculated. Strata were generated based on high (top 40%) and low (lower 40%) community diversity. Survival curves were generated using R packages survival (v 3.2–7) and survminer (v 0.4.9).

### Generalised linear mixed-model analysis

The potential correlation between 16S rRNA gene sequencing data and racing performance was explored using linear mixed-model analysis via glmmTMB and purrr (v 0.3.4) packages in R. Official rating (OR), ranked average race earnings and average race relative-place (1-(race place/number of horses in race)) were used to evaluate racing performance. Similar to the time-to-event analysis, different models were evaluated, based on different sets of predictor variables. Model A included sequencing data and sex; model B included sequencing data, sex, age, birth weight, mare age and gestation days; model C included sequencing data, sex, age, supplemental colostrum (yes/no) and age weaned. OR variation across races within horse is smaller than variation across horses, therefore, a mixed-model including all available OR values per horse was considered and horse id was included as a random effect. For race earnings and relative place, the average of all races per horse was used as the response variable because there was larger variation within horse, therefore, the random effect of horse id was not necessary in the models. Average race earnings were standardised by using ranks. Standardised regression coefficients were obtained by multiplying the model coefficients by the ratio of the independent and dependent variable standard deviations.

The R package ggeffects (v 1.1.4) was used to generate predictions (predicted values and 95% confidence intervals) of the glmm models by holding the covariates constant (numeric values set to mean, factors set to their reference level and character vectors to their mode) and varying the metagenomics variable. The R package prettyGraphics (v 0.1.1) was used to add the regression line (predicted values) and associated error envelope (95% CI) to the scatter plot of metagenomics levels vs performance values.

### Ethics approval and consent to participate

All experimental protocols were approved by the University of Surrey’s NASPA sub-committee under the project reference number NERA-2017–007-SVM. All methods were carried out in full compliance with the University of Surrey Ethics Guide and the Veterinary Surgeons Act (1966). The written informed consent of the owner or manager of each stud farm was obtained prior to commencement of the study.

## Results

### Maturation of gut microbial communities

Bacterial diversity within samples (alpha diversity) was low at early sample points, increasing until 60 days old (SP05). Significant differences in two different metrics of bacterial community diversity (observed bacterial features, and Shannon diversity index) were identified between the first four sample points (one-way ANOVA, Tukey test p < 0.05, Supplementary Fig. 1).

**Figure 1:**
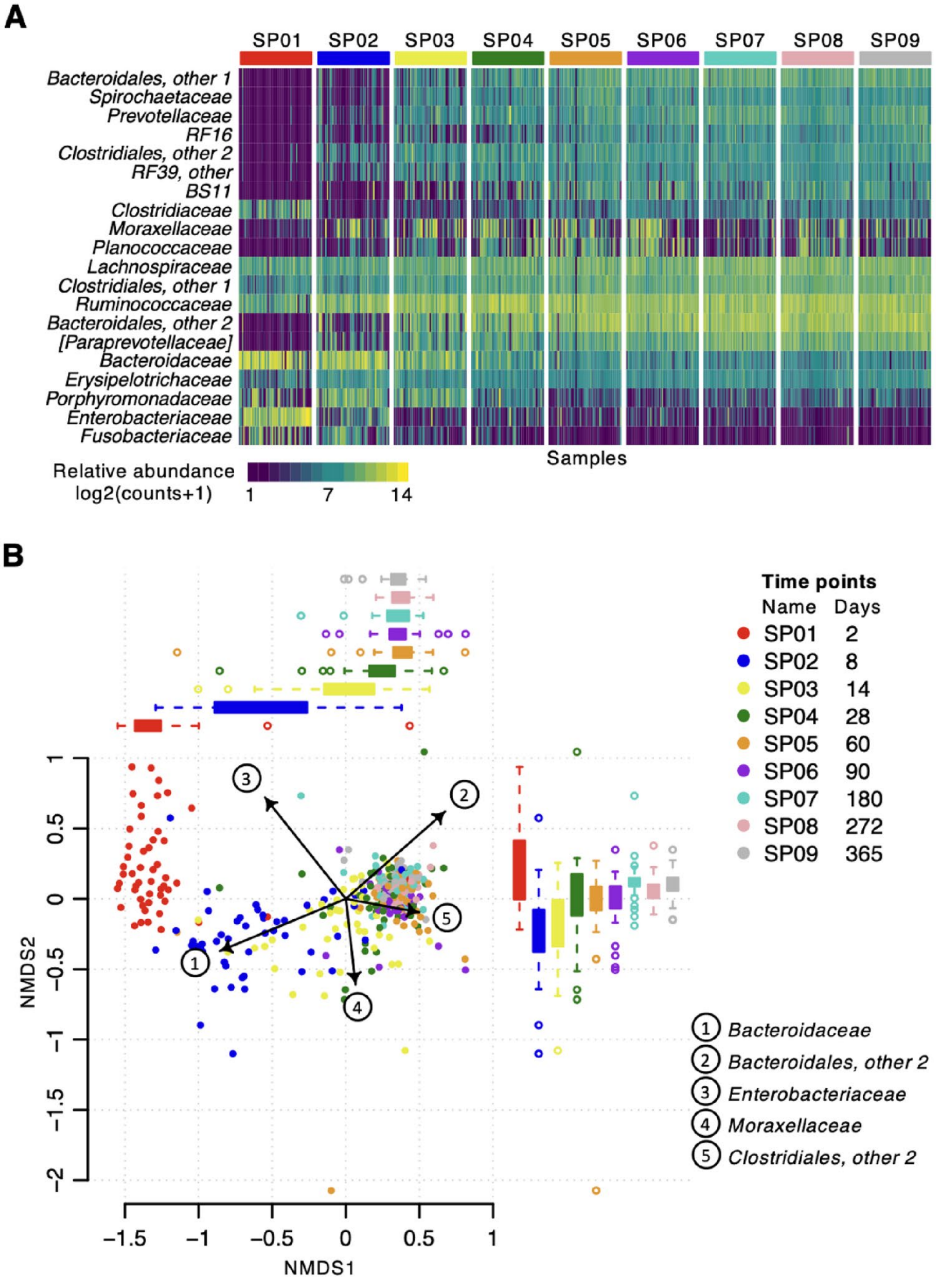
Maturation of the faecal bacterial community during the first year of life in 52 Thoroughbred foals. Data from 16S rRNA sequencing of 430 equine faecal DNA extracts. (**A**) Heatmap for relative abundance of the 20 most abundant bacterial families. Samples grouped by sample point (SP01, 2 days; SP02, 8 days; SP03, 14 days; SP04, 28 days; SP05, 60 days; SP06, 90 days; SP07, 180 days; SP08, 272 days; SP09, 365 days). Abundance scale corresponds to log2(counts + 1), and counts have been rarefied. (**B**) Non-metric multidimensional scaling (NMDS) model of bacterial family abundance. Each point represents a sample, the distance between the points represents the degree of difference, and the horizontal and vertical coordinates represent the relative distance. The arrows indicate bacterial families that covary with the microbial community structure, and thus potentially drive changes within the dataset. The length of the arrow is proportional to the squared correlation coefficient. Horizontal and vertical boxplots show the distribution of the samples on NMDS1 and NMDS2 respectively, grouped by time point, the boundaries of the box represent the 25^th^ and 75^th^ quantiles of the data distribution and whiskers extending from the box denote the minimum and maximum values excluding outliers.

The relative abundance profile of the 30 most abundant bacterial families in foal faecal microbiota during the first year of life is shown in Fig. 1A. The families *Enterobacteriaceae* and *Bacteroidaceae* were most abundant at two days old. Bacteria from the orders *Bacteroidales* and *Clostridiales* were the most abundant in samples from later time points but were variably present, and in low abundance, at early time points. Many bacterial families, including *Spirochaetaceae* and *Fibrobacteraceae*, were not detected at early time points but increased in abundance as the foals aged. The percentage abundance of the ten most common bacterial families across all samples is displayed in Supplementary Fig. 2. Individual variation in bacterial family relative abundance between horses sampled at the same time point is evident and this was most noticeable at early time points.

The identity of bacterial families driving the major changes in gut bacterial community structure during the first year of life was explored using non-metric multidimensional scaling (NMDS, Fig. 1B). Sample proximity in the low dimensional space reflects compositional similarity. Samples collected at 2 and 8 days of age (SP01 and SP02) clustered together and away from the rest of the samples, indicating a markedly different community structure compared to later time points. Vector arrows indicate that this difference is driven by significantly higher relative abundance of *Enterobacteriaceae* and *Bacteroidaceae* at early time points and by *Bacteroidales*, *Moraxellaceae* and *Clostridiales* at later time points.

### Diversity predicts risk of disease

**Figure 2:**
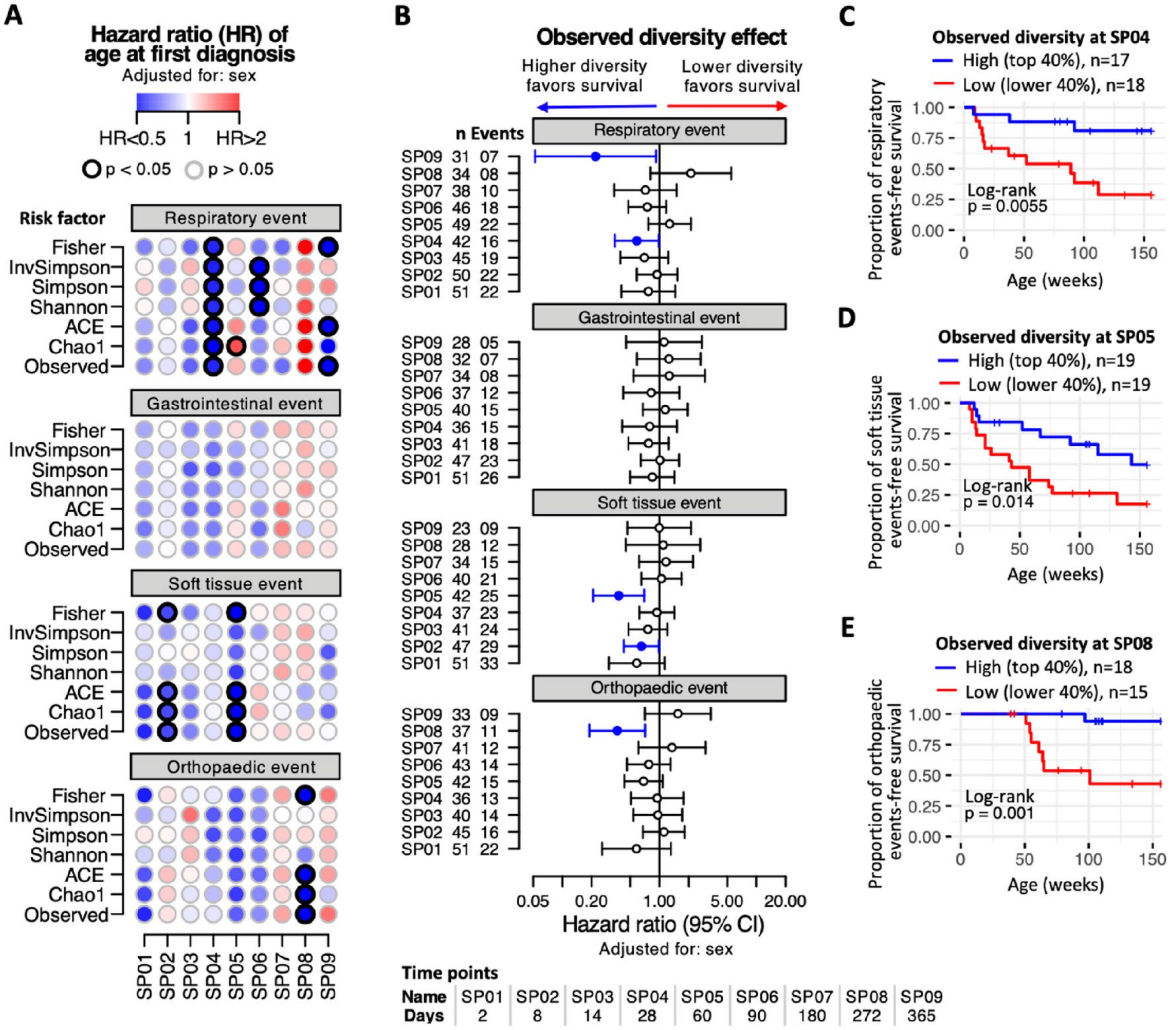
Effect of bacterial diversity on age at first diagnosis. A multivariable Cox mixed-effects model was constructed per sampling point (SP01 to SP09) to assess the relationship between bacterial diversity and age of first diagnosis of a health event. The models were adjusted for sex and stud farm of birth was included as a random effect. Seven different measures of bacterial diversity were explored. (**A**) Time-to-event standardised hazard ratios of respiratory, gastrointestinal, soft tissue or orthopaedic events (row sections), at each of the nine sampling points (horizontal axis), using different diversity measures as risk factors (vertical axis). Each dot corresponds to an independent model. Dot color intensity corresponds to hazard ratio (HR) values, with HR > 1 in red and HR < 1 in blue shades. Dots with a black border line indicate p < 0.05. (**B**) Time-to-event standardized hazard ratios (with 95% confidence interval) when using observed measure of bacterial diversity as risk factor. Vertical axis displays number of horses (n) and number of events per sampling point. Filled circles indicate p < 0.05. (**C**–**E**) Kaplan–Meier time-to-event survival of horses stratified according to observed bacterial diversity as high (top 40%) and low (lower 40%) based on: (**C**) diversity at SP04 (28 days old) as a risk factor for respiratory events, (**D**) diversity at SP05 (60 days old) as risk factor for soft tissue health events and (**E**) diversity at SP08 (272 days old) as risk factor for orthopaedic events.

Using survival analysis, we explored the effect of faecal microbial diversity on the rate at which study subjects developed their first episode of respiratory disease. We found that higher bacterial diversity at 28 days old (SP04) was significantly associated with a reduced risk of respiratory disease later in life (Fig. 2A). This association was present for all seven measures of diversity used. Increases in several diversity measures at 90 days old (SP06) and 365 days old (SP09) were also significantly associated with a reduced risk of respiratory disease. Four survival models were explored, a baseline model adjusted for foal sex, a model adjusted for sex and gestational factors (birth weight), a model adjusted for sex and extended gestational factors (birth weight, mare age and gestation days) and a model adjusted for sex, perinatal factors (supplementary colostrum) and early-life factors (age at weaning) were fitted. All models reported a significant association between some measures of bacterial diversity at 28 days of age and a reduced risk of respiratory disease in later life (Supplementary Fig. 3D). For other health outcomes, we report significantly decreased risk associated with higher faecal bacterial diversity at 28 and 60 days old (soft-tissue disease) and 272 days (orthopaedic disease). Hazard ratios based on observed bacterial diversity are shown in Fig. 2B. Figure 2C–E show the observed effect of low vs high faecal bacterial diversity at different ages (28 days old–272 days old) on various health outcomes. For respiratory, soft-tissue and orthopaedic health events the incidence rate of disease is significantly higher with low gut bacterial diversity in early life.

### Diversity predicts athletic performance

**Figure 3:**
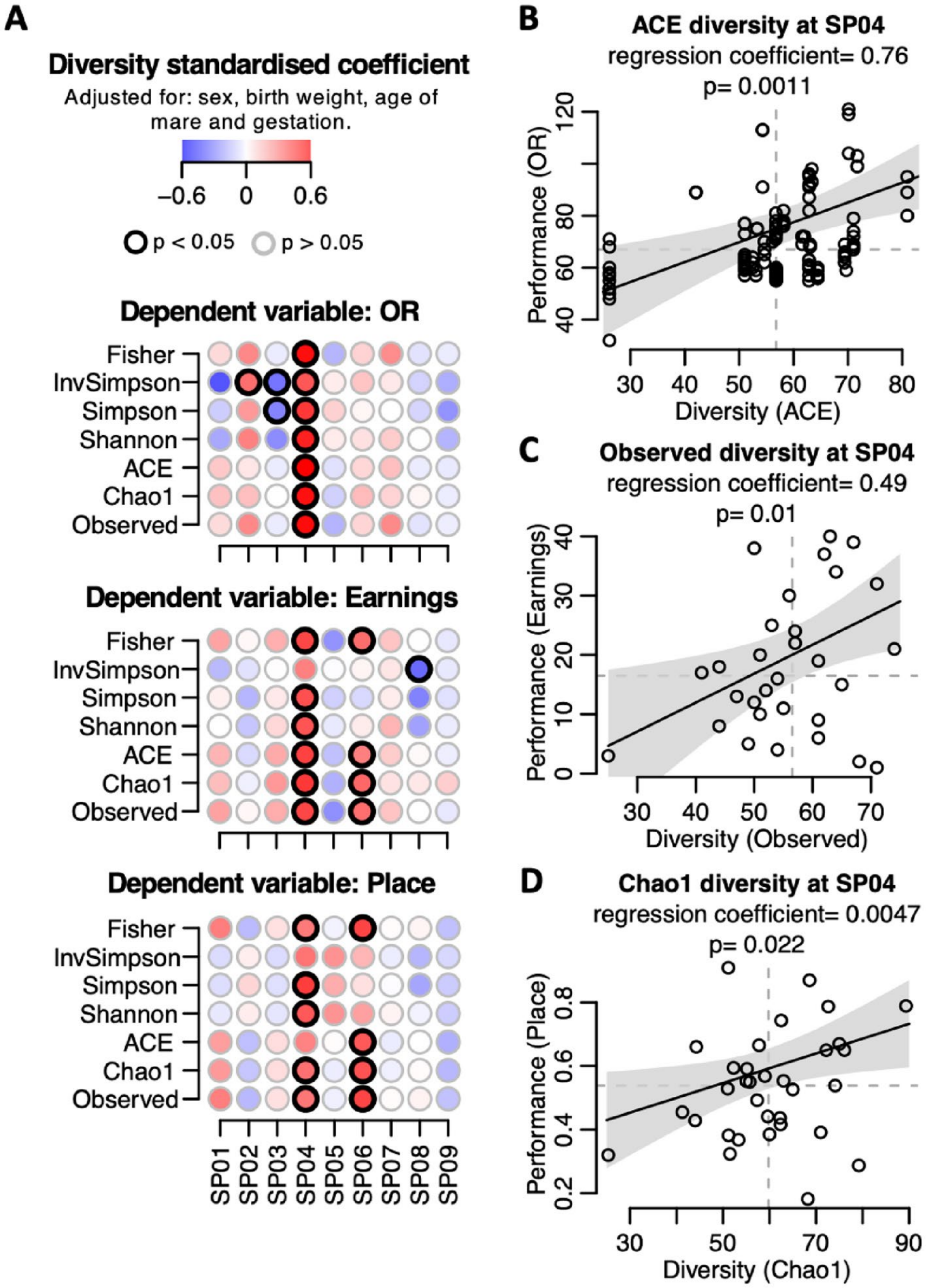
Effect of bacterial diversity on racing performance. A multivariate regression model was constructed per sampling point (SP01 to SP09) to assess the relationship between bacterial diversity and racing performance. The models were adjusted for sex, birth weight, age of mare and gestation. Official rating (OR), earnings and placing metrics were used as proxies for racing performance. (**A**) Diversity standardised regression coefficients for dependant variables OR, earnings and place. Seven different bacterial diversity measures were explored as predictor variables (vertical axis) at each of the nine sampling points (horizontal axis). Each dot corresponds to an independent model. Dot color intensity corresponds to diversity standardized regression coefficients. Dots with a black border line indicate p < 0.05. (**B**–**D**) Performance predicted values (black line, (**B**) OR, (**C**) earnings and (**D**) place) and 95% confidence intervals (grey area) of the regression models when holding the covariates constant (sex, birth weight, age of mare and gestation) and varying the predictor variable: (**B**) ACE diversity, (**C**) observed diversity and (**D**) Chao1 diversity, at SPO4 (28 days old). Circles correspond to data points. Non-standardised regression coefficient and p-value are shown in each panel (**B**–**D**).

Our dataset provided a unique opportunity to explore the influence of early-life gut microbiota on subsequent athletic performance. Generalised linear mixed-models were fitted using three different outcome variables which are proxies for athletic performance: official rating (OR), average prize money earnings and average race placings. We found significant positive associations between faecal microbial diversity at 28 days (SP04) and all three outcome variables, with some measures of diversity at 90 days (SP06) also being significantly associated with performance (Fig. 3A). These regression models controlled for sex, birth weight, age of the mare and gestation days, all recognised confounders of racing performance. More parsimonious models, controlling for fewer confounders, confirmed these findings (Supplementary Fig. 4). In Fig. 3B–D regression lines with 95% confidence envelopes illustrate the relationship between performance data and faecal bacterial diversity at 28 days of age. All three performance outcome measures were significantly and positively associated with gut bacterial diversity at 28 days old.

### Antimicrobial use in early life

**Figure 4:**
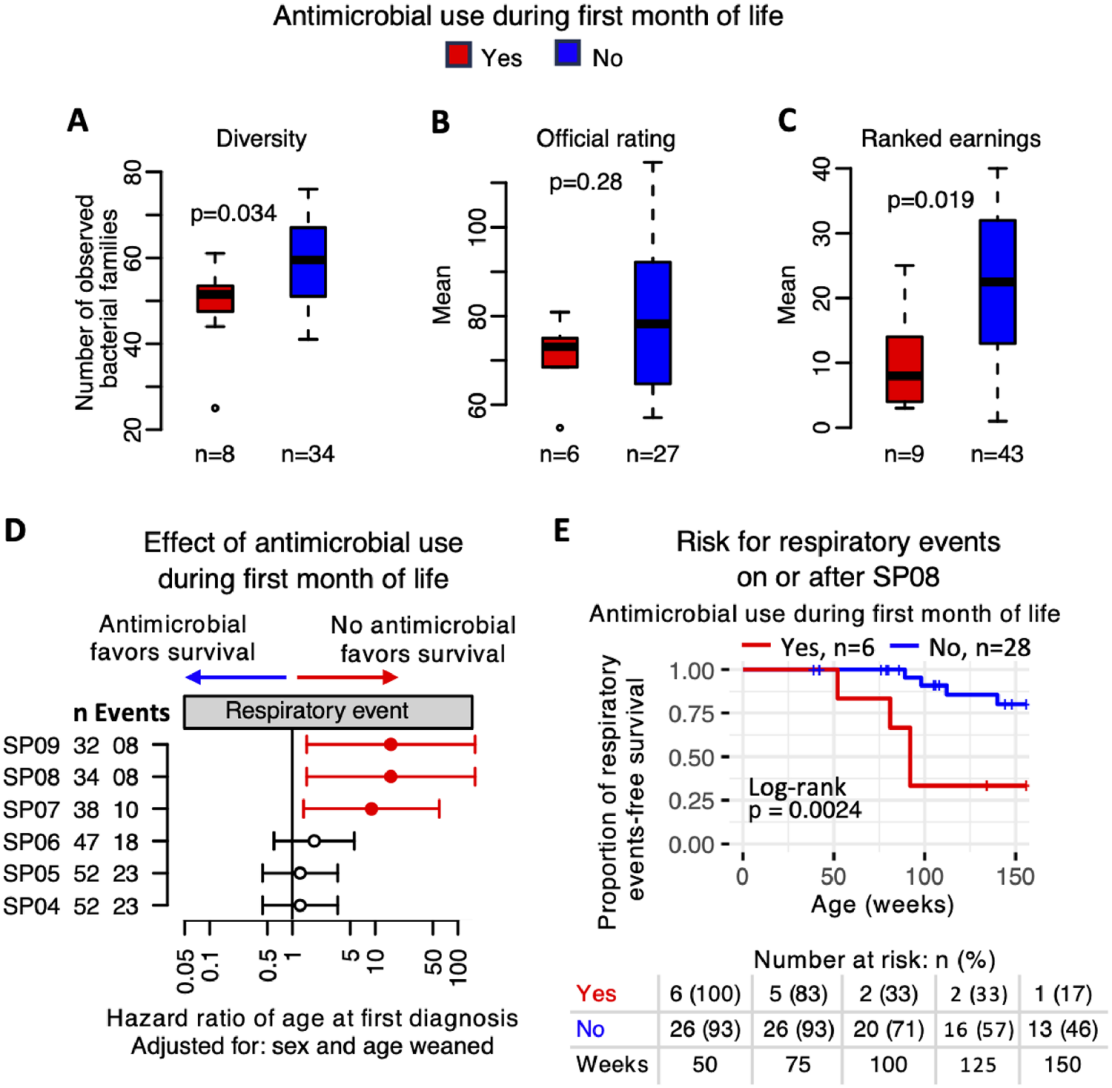
Effect of antimicrobial use during first month of life. (**A**) Comparison of observed diversity of samples taken at SP04 (4 weeks old) from horses that had (Yes) vs did not have (No) antibiotics on or before the sampling point. (**B**) Comparison of mean Official Rating values of horses stratified by antimicrobial use during first month of life. (**C**) Comparison of mean ranked earnings of horses stratified by antimicrobial use during first month of life. For panels (**A**–**C**) box-and-whisker plots where the lower and upper boundaries of the box represent the 25th and 75th quantiles of the data distribution, respectively, the median is indicated by the horizontal line inside the box and whiskers extending from the box denote the minimum and maximum values excluding outliers. All p values are based on a Mann–Whitney test. (**D**) Hazard ratios (with 95% confidence interval) obtained from multivariable Cox mixed-effects models constructed per sampling point (SP04 to SP09) to assess the relationship between antimicrobial use during first month of life and age of first diagnosis of a respiratory health event. The models were adjusted for sex, age weaned, and stud of birth was included as a random effect. Vertical axis displays number of horses (n) and number of events per sampling point. Filled circles indicate p < 0.05. (**E**) Kaplan–Meier time-to-event survival (risk of respiratory events on or after SP08) of horses stratified according to antimicrobial use during first month of life as ‘Yes’ or ‘No’.

The study population was further categorised according to early-life exposure to antimicrobial medication. Figure 4 illustrates that foals receiving antimicrobial treatment during the first month of life had significantly lower faecal bacterial diversity at 28 days old (Fig. 4A, Mann–Whitney test, *p* < 0.05). In their subsequent racing careers (up to 3 years of age) average prize money earnings (a proxy for athletic performance) was significantly lower in foals subjected to this exposure (Fig. 4C, Mann–Whitney test, *p* < 0.05) and the distribution of official ratings differed according to antimicrobial exposure (Fig. 4B), but this was not statistically significant. Survival analysis identifies a significantly increased rate of respiratory disease in foals that had received antimicrobial treatment before 28 days of age (Fig. 4D and E). Supplementary Fig. 5 illustrates the impact of early life antimicrobial administration on gastrointestinal, soft tissue and orthopaedic health outcomes, none of which were statistically significant. NMDS modelling of changes in community profile associated with antimicrobial treatment (Supplementary Fig. 6) identifies the bacterial families *Bacteroidales* and *Xanthomondaceae* as covarying with antimicrobial exposure and therefore being potential drivers of change.

### Bacterial community structure at 28 days

The impact of gut bacterial diversity in foals at 28 days old on health and performance in later life led us to explore bacterial community structure at this age in more detail. Foals were categorised as high, middle or low diversity based on the Shannon diversity measure of each sample at 28 days old (SP04). When categorised in this way the three groups of horses had significantly different Shannon diversity to one another (one-way ANOVA, Tukey test *p* < 0.05, Supplementary Fig. 7A). Low and middle diversity samples were dominated by bacteria from the *Ruminococcaceae* (on average 21% of total reads), although this did vary between individuals (Supplementary Fig. 7B). Many of the samples with low diversity comprised over 40% of a single bacterial family. NMDS analysis of the sequence counts identified *Bacteroidales*, *Prevotellaceae* and *Planococcaceae* as covarying with community structure (Supplementary Fig. 7C), indicating their potential roles as drivers of change.

### Bacterial families and disease risk

We used survival analysis to identify bacterial families that were significantly associated with illness or injury later in life (Supplementary Fig. 8). Respiratory disease was significantly and positively associated with the relative abundance of faecal *Pseudomonadaceae* at 8 days old (SP02). Soft tissue injury or disease was positively associated with the faecal relative abundance of *Streptococcaceae* at 8 days old (SP02) and *Moraxellaceae* at 14 days old (SP03); gastrointestinal illness and disease was associated with the relative abundance of faecal *Rikenellaceae* at 90 days old (SP06).

### Bacterial families and racing performance

Using three different measures of athletic performance in generalized linear mixed models with bacterial family abundance as the explanatory variable, we identified bacterial families that were significantly and positively associated with performance outcomes (Supplementary Fig. 9). Across all three models run with faecal bacterial community data from foals at 28 days of age (SP04), higher relative abundance of *Anaeroplasmataceae* was associated with higher official rating (OR, Supplementary Fig. 9A) and higher relative abundance of *Bacillaceae* was associated with higher race placings (Supplementary Fig. 9C). Some associations were identified by only one model: higher abundance of an unknown bacterial family belonging to the phylum OD1 at 272 days old (SP07) was associated with higher OR, higher abundance of *BS11* at 28 days old (SP04) was associated with higher earnings and higher abundance of *Coriobacteriaceae* at 60 days old (SP05) was associated with higher placings. Regression plots, with 95% confidence envelopes, illustrate the relationship between performance metrics and abundance of *Anaeroplasmataceae*, *BS11* and *Bacillaceae* (Supplementary Fig. 9D–F).

## Discussion

This study has identified significant associations between gut microbial community structure in horses at a surprisingly young age and both health and athletic performance outcomes up to three years later in life. We have also identified associations between specific bacterial taxa, specific health outcomes and measures of athletic performance.

Our observational study identifies associations between outcomes and explanatory variables but the study design does not allow the attribution of causality. However, some features of the observed associations are consistent with observed relationships being causal^23,24^: (i) the “strength”/magnitude of the associations, (ii) consistency (e.g. with different survival models and different outcome measures for athletic performance), (iii) the specificity for certain disease outcomes, (iv) temporality of the relationship, (v) biological gradient (e.g. enhanced performance with higher diversity, Fig. 3) and (vi) biological plausibility. The impact of early-life antimicrobial exposure (an intervention) on subsequent risk of respiratory disease and athletic performance provides additional support for the causal nature of the observed associations. We contend that a causal relationship is a plausible explanation for our findings.

The existence of a “critical window,” a period early in life when gut microbial community structure imprints immunity and future health outcomes, is supported by experimental animal studies^25^, longitudinal studies of childhood health^26–34^ and indirectly by studies of the impact of early life antibiotic use on human health outcomes^35^. In the current study, we identified a “critical window” for respiratory disease risk in horses at around 28 days of age. Our data modelling also provides some evidence of association between diversity at 14—60 days of age and other health outcomes (orthopaedic, soft tissue and gastrointestinal) although the signal is less consistent.

It is unclear why gut microbial communities at 28 days old are so influential on future respiratory health and athletic performance. Environmental changes around this time include increasing exposure to pasture (with associated grass and soil microbiota) and an increasing tendance for foals to eat their mother’s diet in addition to suckling. Immunologically, a foal’s passive immunity is waning during the first weeks of life. Maternally-derived immunoglobulin G from colostrum has a half-life of 18–32 days; by 28days old passively derived antibodies have declined by over 50% but autogenous immunoglobulin production does not mature until 90 days old^36,37^. The dynamics of neonatal immunological development may be significant in the timing of the critical window.

The relationship between gut bacterial diversity at one month old, antibiotic treatment and respiratory disease in later life could potentially be confounded by the disease that was the cause of antibiotic treatment. However, this is not the case in our dataset as none of the foals receiving antimicrobials during the first month of life were treated for respiratory disease.

A “weaning reaction,” characterised as a period of immunological reactivity associated with weaning, has been described in mice at 3 weeks of age^38^. In neonatal pigs, a pre-weaning (i.e. before 28 days old) window for modulation of gut microbiota and the immune system has been described^39^. There is less clarity around the timing of the critical window in human infants, although most studies infer that it exists during the pre-weaning period of infancy i.e. between birth and approximately 6 months of age^40^. In our cohort of foals weaning typically occurred around 5 months of age; our study suggests that for horses, and for some health and performance outcomes, critical windows occur long before weaning.

The influence of diet and immunological modulation on athletic performance have been explored previously^41–43^, but our study is the first to demonstrate the impact of early-life gut microbiota. Our findings introduce a new and important concept to the emerging science of sportomics^44^. Studies to date have focussed on real-time metabolic interactions between athletes and their gut microbiome^45^, including equine athletes^46^. We demonstrate that gut microbial diversity in early-life (1 month old) predicts athletic performance in later life.

Identification of an association between the enteric abundance of *Pseudomonadaceae* and adverse respiratory health events is interesting. *Pseudomonas aeruginosa*, a member of this bacterial family, is frequently isolated from the lungs of foals with pneumonia^47^, but why should it also be abundant in the hind gut/faecal microbiota? We postulate that oro-pharyngeal colonization leads to seeding of *Pseudomonadaceae* in both lungs and the hindgut. This hypothesis is supported by oropharyngeal colonization of *Pseudomonadaceae* being a risk factor for ventilator-associated pneumonia in human critical care patients^48^. We also identified a positive association between the faecal abundance of both *Streptococcaceae* and *Moraxellaceae*, and the risk of soft-tissue health events (e.g. infected wounds, cellulitis and abscesses). *Streptococcaceae* are frequently isolated from equine soft-tissue lesions^49^; infection with *Moraxellaceae* is less well characterised in horses but in humans is associated with infection at a range of sites^50^. Perhaps the co-occurrence of a specific bacterial family in the hindgut microbiota and at a site of clinical infection is the result of seeding, to both the gut and the infection site, from an established reservoir elsewhere in the animals e.g. the mouth^51^ or oropharynx?

Limitations of our study include the taxonomic resolution possible with 16S rRNA gene sequencing data, the granularity of health event data and the imprecise nature of measures of racehorse athletic performance^52^. Exploration of the impact of antimicrobial treatment on our study cohort was not a primary objective and this study which was not powered to detect such effects. Interpretation of data on antimicrobial use should be done with the understanding that study power is low (due to the small number of observations) with a relatively high probability of type II error. A further limitation is the absence of data on mare-associated factors that might influence colonisation of the neonatal foal gut. The gut bacterial community profile of human mothers is known to be a primary driver of gut microbial colonisation in human infants^53–55^. Understanding how the dam’s diet, health history and milk microbial composition are associated with foal gut colonisation and health outcomes are worthy of further study.

## Conclusion

Using data from a carefully phenotyped cohort of Thoroughbred foals, we have described colonisation and maturation of the gut microbiota and revealed associations between early-life bacterial community profiles, health events and athletic performance in later life. Gut bacterial diversity at 28 days old predicts the risk of respiratory disease in later life and we identify bacterial families that are associated with subsequent health and athletic performance. Our study provides evidence for a detrimental association between antimicrobial treatment in the first month of life and health outcomes and performance outcomes in later life.

These findings provide an evidence-base for early-life interventions designed to enhance the health and future athletic performance of horses bred for racing including, where possible, the avoidance of antimicrobial therapy. For the first time, this study demonstrates a relationship between early-life gut bacterial communities and subsequent athletic performance that has implications for athletes of all species including humans.

### Supplementary Information

Supplementary Information.

## Data Availability

All 16S rRNA gene sequence data can be accessed on the Sequencing Read Archive under BioProject ID PRJNA812865. Health and performance data are not publicly available for privacy reasons but are available from the corresponding author on reasonable request. Pre-processing, analyses, and result plots were generated using QIIME2, a cutting-edge microbiome bioinformatics platform, in conjunction with R, a robust environment for statistical computing and graphics. Both tools are freely available and open source. Details of the specific QIIME2 and R functions employed are outlined in the methods section.
