# Supplementary material for: Early-life gut bacterial community structure predicts disease risk and athletic performance in horses bred for racing
Source: Sci Rep. 2024 Aug 7;14:17124. doi: 10.1038/s41598-024-64657-6 (PMC11306797; doi:10.1038/s41598-024-64657-6)
Supplement: Supplementary file 1 — Supplementary Information. [file 41598_2024_64657_MOESM1_ESM.docx]

**SUPPLEMENTARY INFORMATION**

**Supplementary table 1:** Further information on the foals enrolled onto the study. Ortho, orthopaedic disease or injury; soft, soft tissue disease or injury; GI, gastrointestinal illness; resp, respiratory illness. Five foals with less than 52 weeks of data (foals 01, 06, 26, 46 and 54) were excluded from analysis.

| **Foal ID** | **DOB** | **Sex** | **Stud** | **Health summary year1: week(s) and illness/injury** | **Final health update (age in weeks)** |
| --- | --- | --- | --- | --- | --- |
| 01 | 08.02.2018 | M | A | 1 - GI | 33 |
| 02 | 13.02.2018 | M | A | 21 - soft | 186 |
| 03 | 18.02.2018 | M | E | None | 174 |
| 04 | 04.03.2018 | F | E | None | 185 |
| 05 | 04.03.2018 | M | E | None | 120 |
| 06 | 06.03.2018 | M | A | 1, 2 - ortho  10 - soft  17 - resp | 39 |
| 07 | 07.03.2018 | F | E | 6, 21 - soft | 80 |
| 08 | 20.03.2018 | M | C | 1 - ortho | 131 |
| 09 | 20.03.2018 | F | C | None | 185 |
| 10 | 15.03.2018 | M | A | 3, 5, 29 - GI  15 - resp  24 - soft  41- soft  51 - ortho | 185 |
| 11 | 20.03.2018 | F | C | None | 182 |
| 12 | 23.03.2018 | M | C | 1 - ortho | 108 |
| 13 | 25.03.2018 | F | C | 27, 28 - GI | 111 |
| 14 | 24.03.2018 | F | A | 1, 2, 8 -GI  37 - resp | 185 |
| 15 | 27.03.2018 | M | D | 10-12, 27, 31 - soft  16-25 – ortho | 168 |
| 17 | 29.03.2018 | M | D | 8, 9, 18 - GI  21 - resp | 168 |
| 18 | 31.03.2018 | M | D | 2, 3 - soft  10-12 - ortho  13-15 - resp | 79 |
| 22 | 01.04.2018 | M | D | 21 - resp | 168 |
| 23 | 02.04.2018 | M | D | 3-5 - soft  22 - soft | 160 |
| 24 | 02.04.2018 | F | D | 17 - GI  22 – soft  41 - ortho  43 - soft | 144 |
| 25 | 30.03.2018 | M | A | 3, 29, 32 - soft  11-14 - resp | 166 |
| 26 | 01.04.2018 | M | A | 1-3 - GI  4- soft  16 - soft | 23 |
| 27 | 05.04.2018 | M | D | 1 - ortho  7, 17 - GI | 167 |
| 28 | 05.04.2018 | F | D | 1, 11-13, 39 - soft | 134 |
| 29 | 09.04.2018 | M | E | 10 - resp  12-14 - ortho  16, 18- soft | 76 |
| 30 | 08.04.2018 | M | D | 10 - GI | 167 |
| 31 | 10.04.2018 | F | C | 4 - ortho  8 - soft | 179 |
| 32 | 11.04.2018 | F | D | 9 - resp | 166 |
| 33 | 10.04.2018 | F | E | 26 - soft  46 - GI | 79 |
| 35 | 10.04.2018 | M | B | 2, 3 - soft  4-7 - GI  16 - resp | 108 |
| 36 | 13.04.2018 | M | D | 21, 22 - soft  52 - GI | 166 |
| 37 | 13.04.2018 | F | D | 1, 10 - soft  42 - GI | 148 |
| 38 | 15.04.2018 | M | D | 3, 4, 43, 47 - GI  52 - soft | 166 |
| 39 | 16.04.2018 | F | C | 31 - ortho | 179 |
| 40 | 13.04.2018 | M | B | 21-25 - resp | 108 |
| 41 | 13.04.2018 | M | B | 16 - soft  17-19 - GI  25, 26 - resp | 108 |
| 42 | 21.04.2018 | F | D | 38 - resp | 94 |
| 43 | 21.04.2018 | F | C | 17 - soft | 177 |
| 44 | 23.04.2018 | F | B | 9, 10 - resp | 106 |
| 45 | 24.04.2018 | M | D | 4- soft  26, 38-40 - soft | 164 |
| 46 | 24.04.2018 | M | E | 6- GI  8, 9 - resp | 31 |
| 47 | 28.04.2018 | F | C | None | 176 |
| 48 | 28.04.2018 | M | B | 1 – soft  2, 3, 8 -ortho  4 - GI | 106 |
| 49 | 29.04.2018 | F | B | None | 106 |
| 50 | 01.05.2018 | F | A | 2 - GI  8 - soft  18 - soft | 176 |
| 51 | 04.05.2018 | M | B | 38-45 – soft | 105 |
| 52 | 05.05.2018 | M | B | 1 - soft  13, 14 - GI | 105 |
| 53 | 04.05.2018 | F | B | 13, 14, 24 - GI  16 - soft | 105 |
| 54 | 08.05.2018 | M | E | None | 29 |
| 55 | 03.05.2018 | M | E | 2-5 - soft | 110 |
| 56 | 02.05.2018 | F | E | 13 - soft | 76 |
| 57 | 10.05.2018 | M | C | 1-4 - ortho  11 - GI  52 - resp | 162 |

**
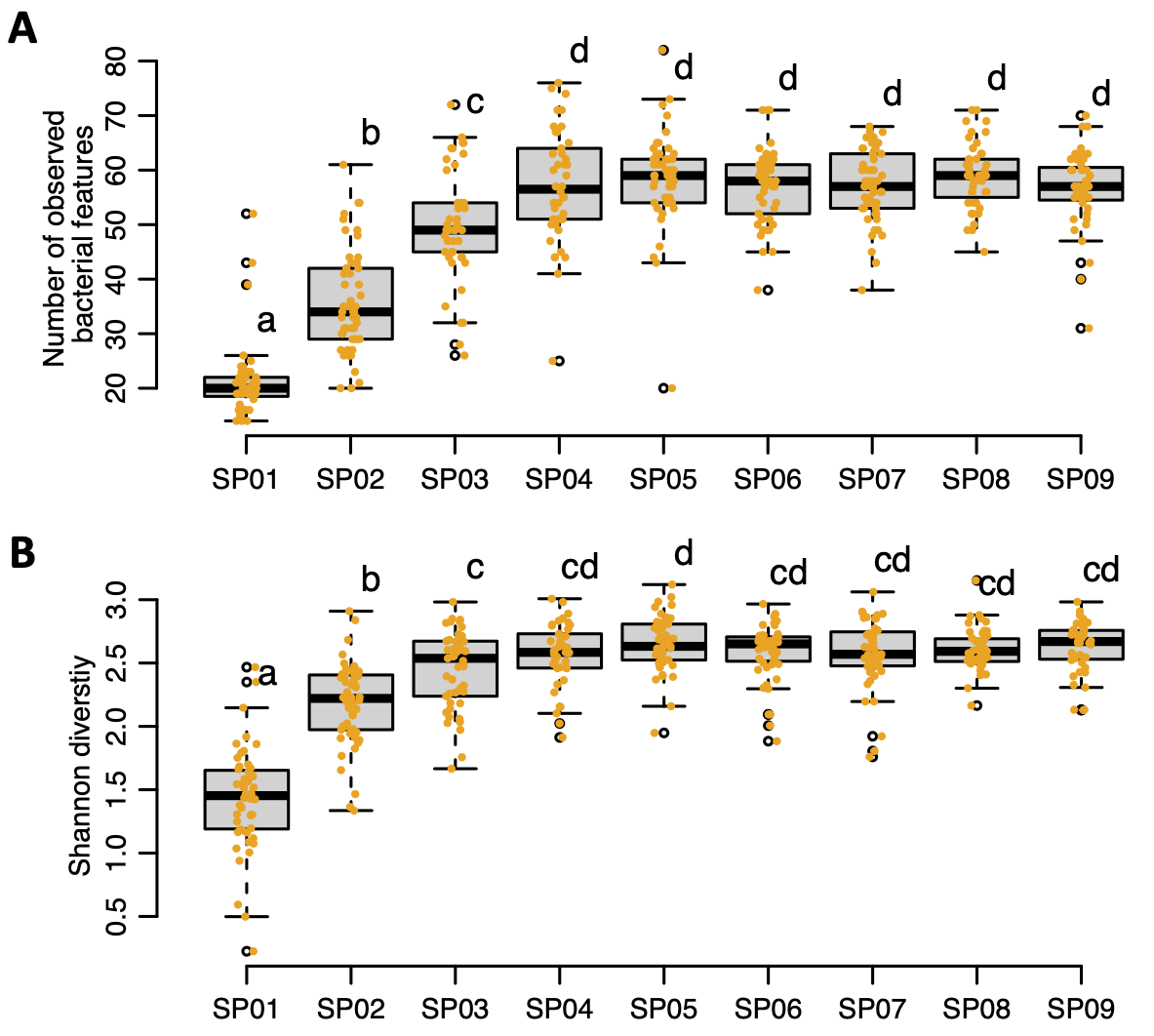
**

**Supplementary figure 1:** Box pots showing the bacterial diversity per sample when samples were grouped by sample point measured as: **A**) Number of observed bacterial features and **B**) Shannon diversity. The lower and upper boundaries of the box represent the 25^th^ and 75^th^ quantiles of the data distribution, respectively, while the median is indicated by the horizontal line inside the box. The whiskers extending from the box denote the minimum and maximum values excluding outliers. Different letters above the boxplots indicate a significant difference between the bacterial diversity of the sample points (One-way ANOVA, Tukey test p<0.05); common characters identify groups that are not significantly different.

**
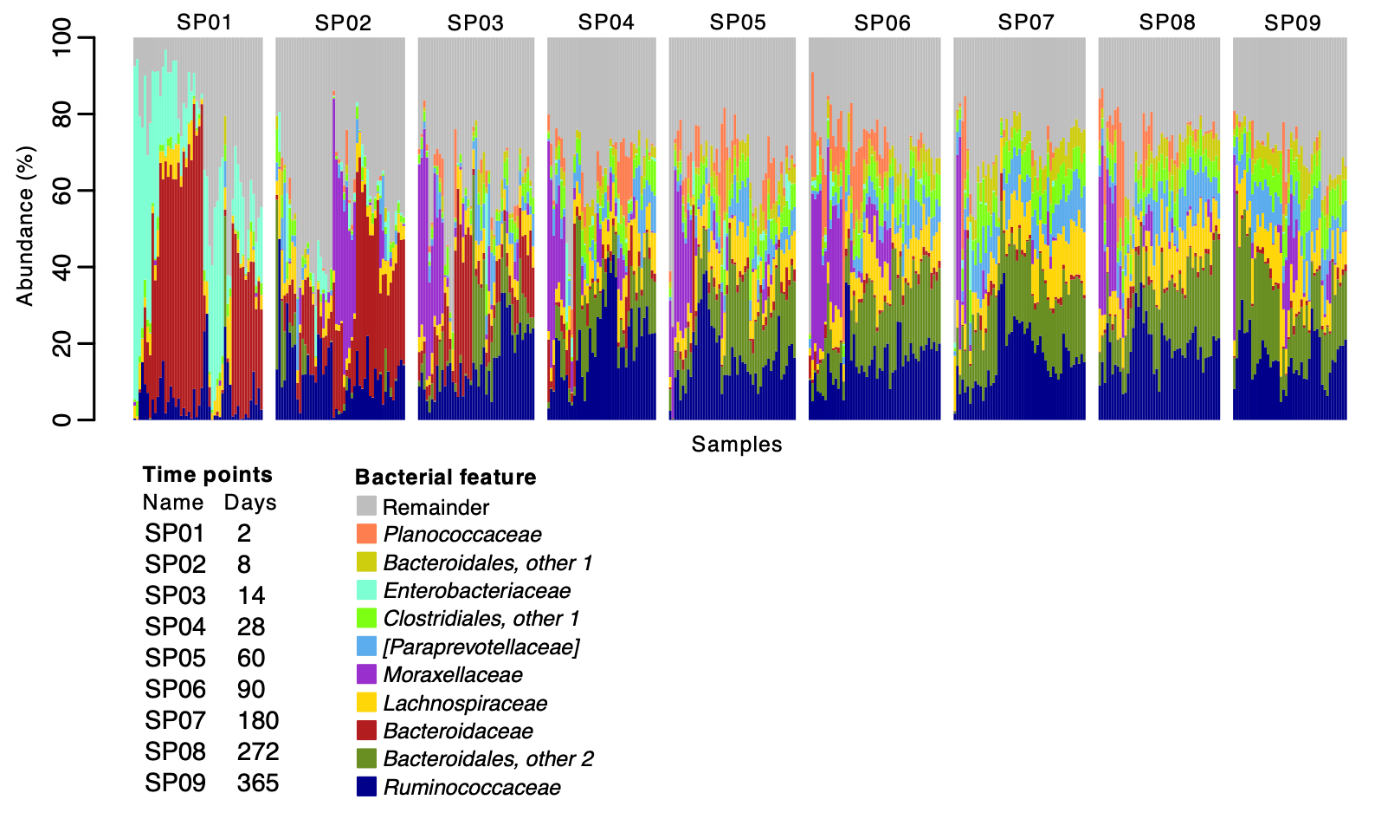
**

**Supplementary figure 2:** Bar plot of relative abundance of the 10 most abundant bacterial families in the dataset. Samples are grouped by sample point. The percentage abundance for all other bacterial families is grouped into the “Remainder” group.


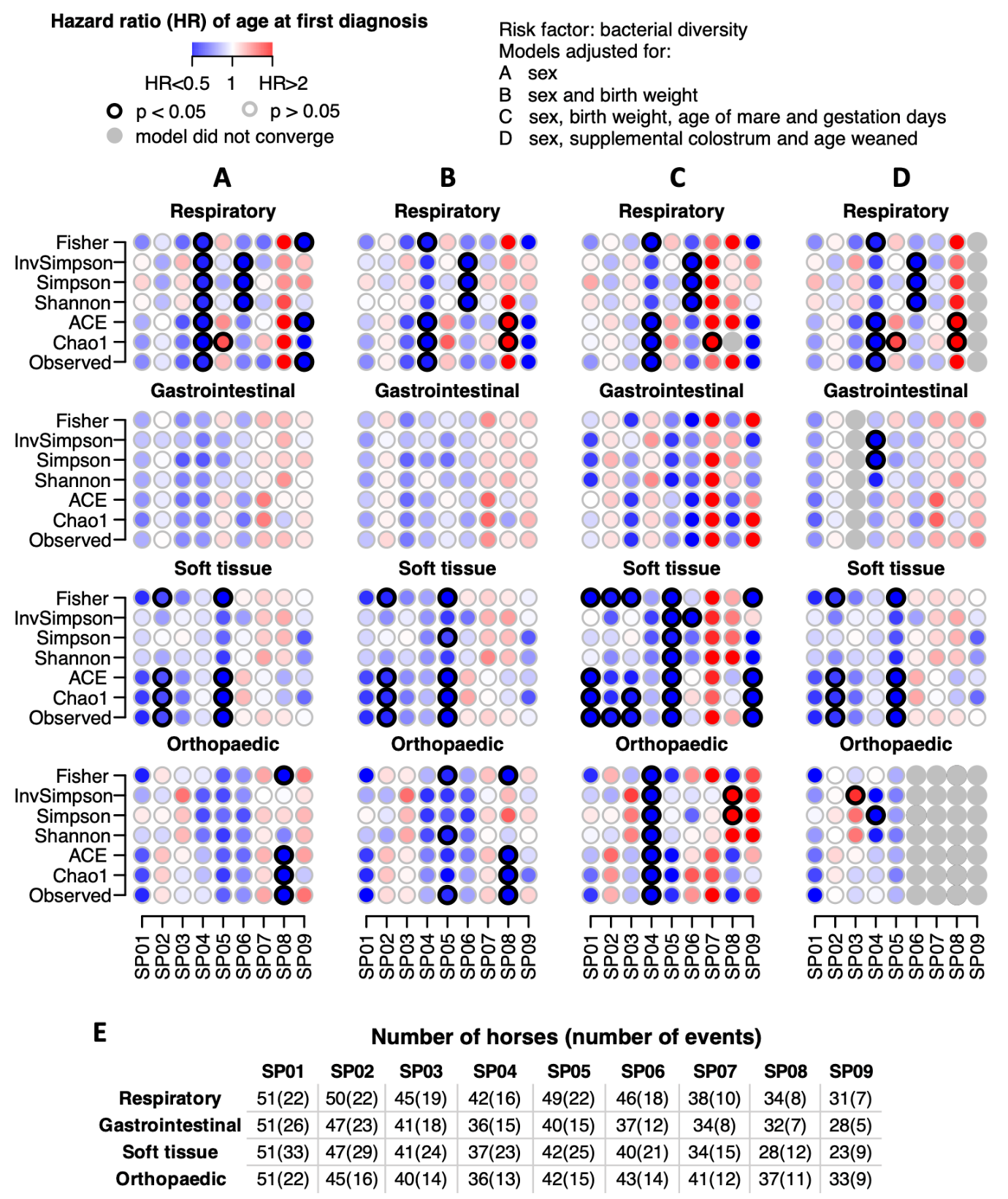


**Supplementary figure 3:** Effect of bacterial diversity on age at first diagnosis. A multivariable Cox mixed-effects model was constructed per sampling point (SP01 to SP09) to assess the relationship between bacterial diversity and age of first diagnosis of a health event. The models were adjusted for different sets of variables (panels A-D) and stud of birth was included as a random effect. Seven different bacterial diversity measures were explored. **A-D** Dot plots show time-to-event standardised hazard ratios of respiratory, gastrointestinal, soft tissue or orthopaedic events (row sections), at each of the nine sampling points (horizontal axis), using different diversity measures as risk factors (vertical axis). Each dot corresponds to an independent model. Dot color intensity corresponds to hazard ratio (HR) values, with HR>1 in red and HR<1 in blue shades. Dots with a black border line indicate p<0.05. **E** Table of number of horses and number of health events in each model.

**
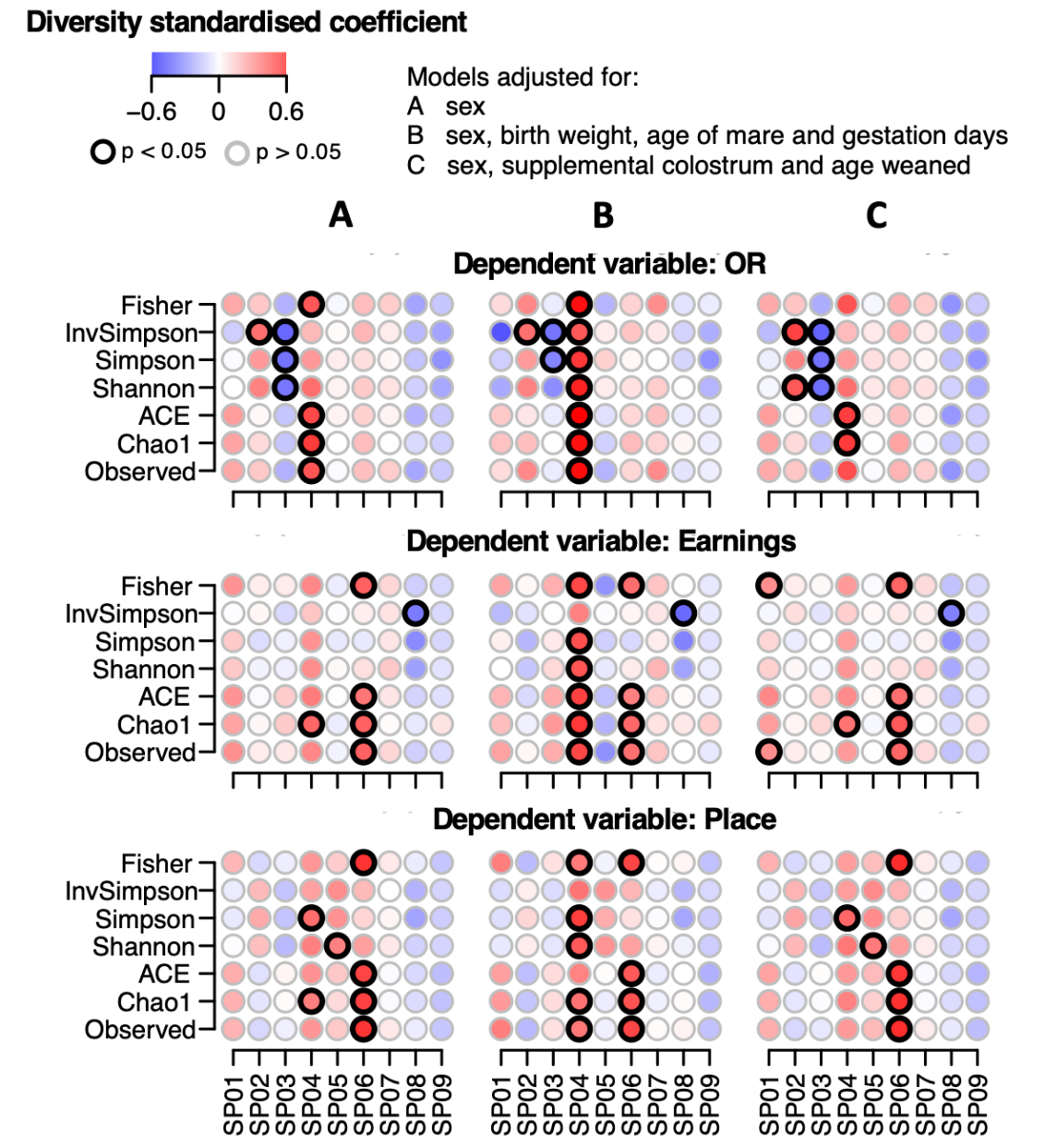
**

**Supplementary figure 4:** Effect of bacterial diversity on racing performance. A multivariate regression model was constructed per sampling point (SP01 to SP09) to assess the relationship between bacterial diversity and racing performance. The models were adjusted for different sets of variables (panels A-C). Official rating (OR), earnings and placing measures were used as proxy for racing performance. **A-C** Dot plots show diversity standardised regression coefficients for dependant variables OR, earnings and place. Seven different bacterial diversity measures were explored as predictor variables (vertical axis) at each of the nine sampling points (horizontal axis). Each dot corresponds to an independent model. Dot color intensity corresponds to diversity standardized regression coefficients. Dots with a black border line indicate p<0.05.

**
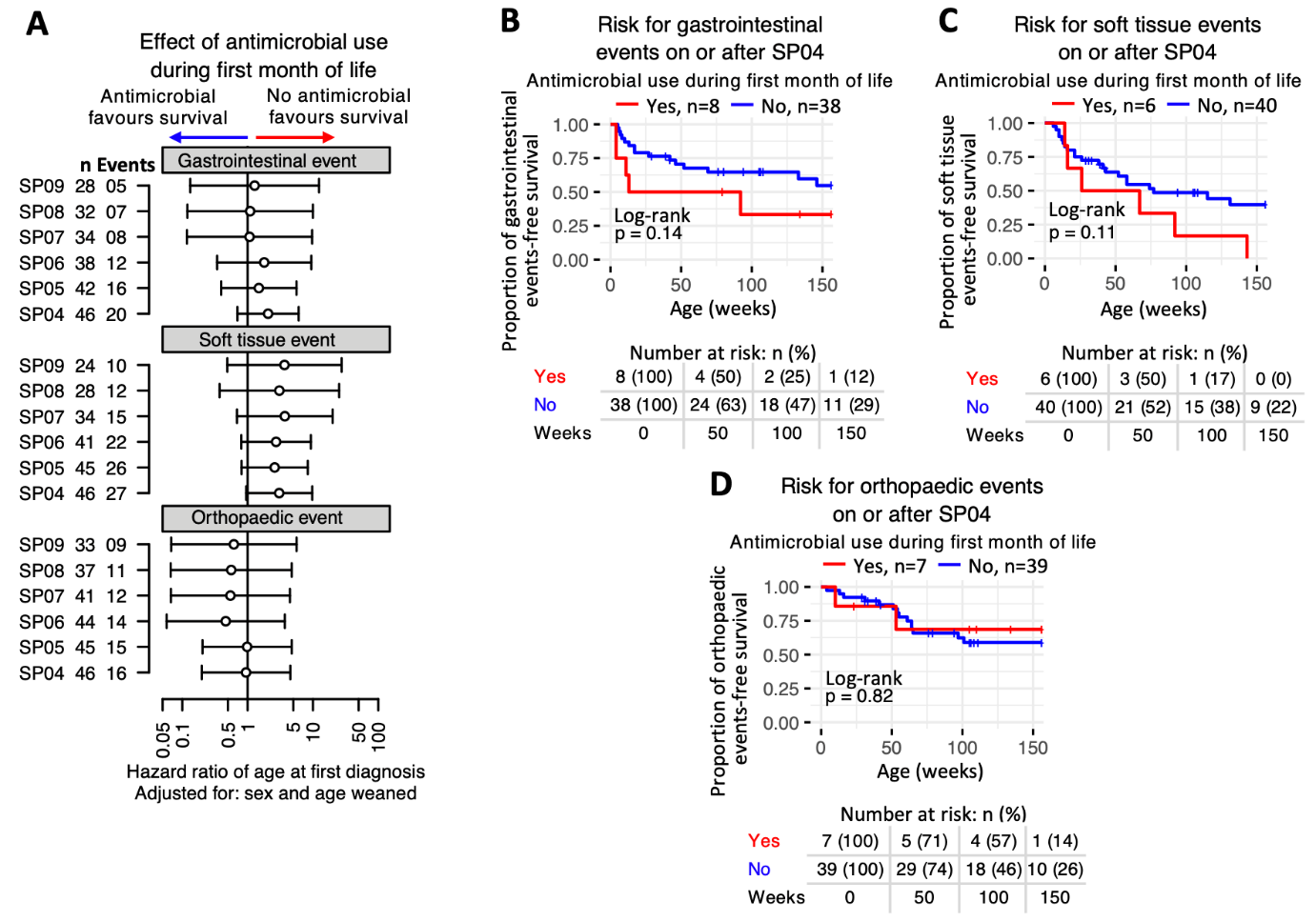
**

**Supplementary figure 5:** Association between antimicrobial use during first month of life and gastrointestinal, soft-tissue and orthopaedic health events. **A)** Hazard ratios (with 95% confidence interval) obtained from multivariable Cox mixed-effects models constructed per sampling point (SP04 to SP09) to assess the relationship between antimicrobial use during first month of life and age of first diagnosis of a health event. The models were adjusted for sex, age weaned, and stud of birth was included as a random effect. Vertical axis displays number of horses (n) and number of events per sampling point. All hazard ratios have p > 0.05. **B-D)** Kaplan-Meier time-to-event survival of horses stratified according to antimicrobial use during first month of life as ‘Yes’ or ‘No’ based on **B** risk for gastrointestinal events on or after SP04, **C** risk for soft tissue events on or after SP04 and **D** risk for orthopaedic events on or after SP04.

**
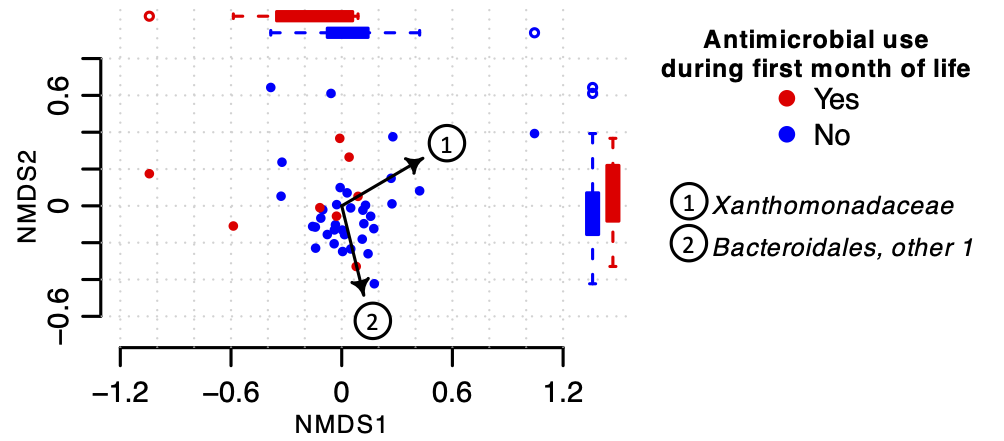
**

**Supplementary figure 6:** Non-metric multidimensional scaling (NMDS) model of bacterial family abundance data from samples collected at 28 days (SP04). Each point represents a sample. Samples are coloured by whether the foal received antibiotics in their first month of life. The distance between the points represents the degree of difference, and the horizontal and vertical coordinates represent the relative distance. The arrows indicate bacterial families that covary with the microbial community structure, and thus potentially drive changes within the dataset. The length of the arrow is proportional to the squared correlation coefficient. Horizontal and vertical boxplots show the distribution of the samples on NMDS1 and NMDS2 respectively, grouped by antimicrobial use during first month of life (yes or no). The boundaries of each box represent the 25^th^ and 75^th^ quantiles of the data distribution and whiskers extending from the box denote the minimum and maximum values excluding outliers.


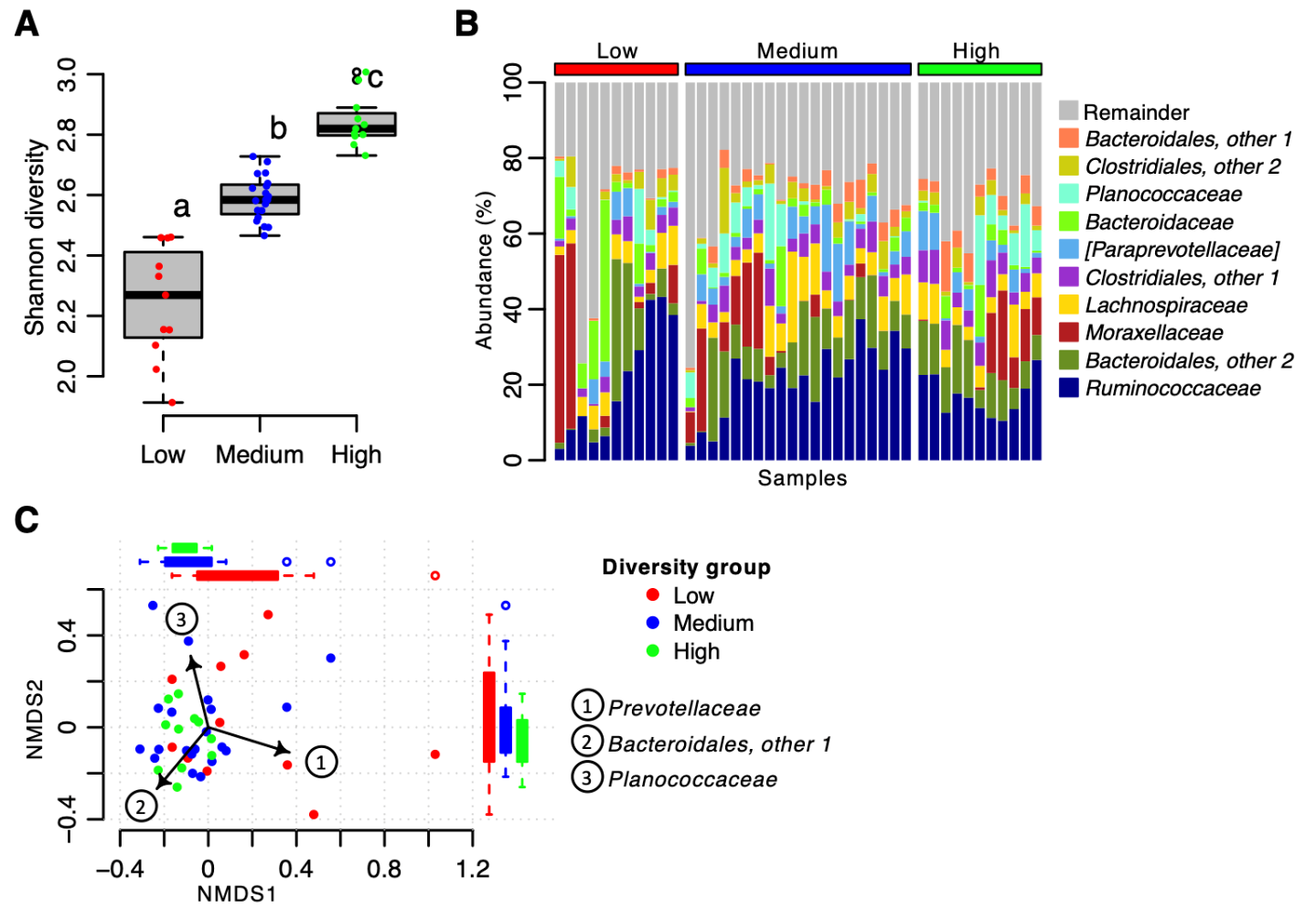


**Supplementary figure 7:** Faecal microbiota at 28 days old (SP04) for foals grouped according to faecal bacterial diversity as “high” (top quartile, n=11), “low” (lower quartile, n=11), and “medium” (remainder, n=20). **A**) Box plots of Shannon diversity per group. The lower and upper boundaries of the box represent the 25^th^ and 75^th^ quantiles of the data distribution respectively, while the median is indicated by the horizontal line inside the box. The whiskers extending from the box denote the minimum and maximum values excluding outliers. Different letters above the boxplots indicate a significant difference between the groups (One-way ANOVA, Tukey test p<0.05); common characters identify groups that are not significantly different. **B)** Bar plot of relative abundance of the 10 most abundant bacterial families in samples taken at SP04. Samples are grouped by their diversity group (high, medium, or low). The percentage abundance for all other bacterial families is grouped into the “Remainder” group. **C)** NMDS model built with the abundance of bacterial families in samples at 28 days old. Only families present in at least 10 samples were used to build the model. Each point represents a sample. Samples are coloured by their diversity group (high, medium, or low). The distance between the points represents the degree of difference, and the horizontal and vertical coordinates represent the relative distance. The arrows indicate bacterial families that covary with the microbial community structure, and thus potentially drive changes within the dataset. The length of the arrow is proportional the squared correlation coefficient. Horizontal and vertical boxplots show the distribution of the samples on NMDS1 and NMDS2, respectively, grouped by diversity category (low, medium, high).


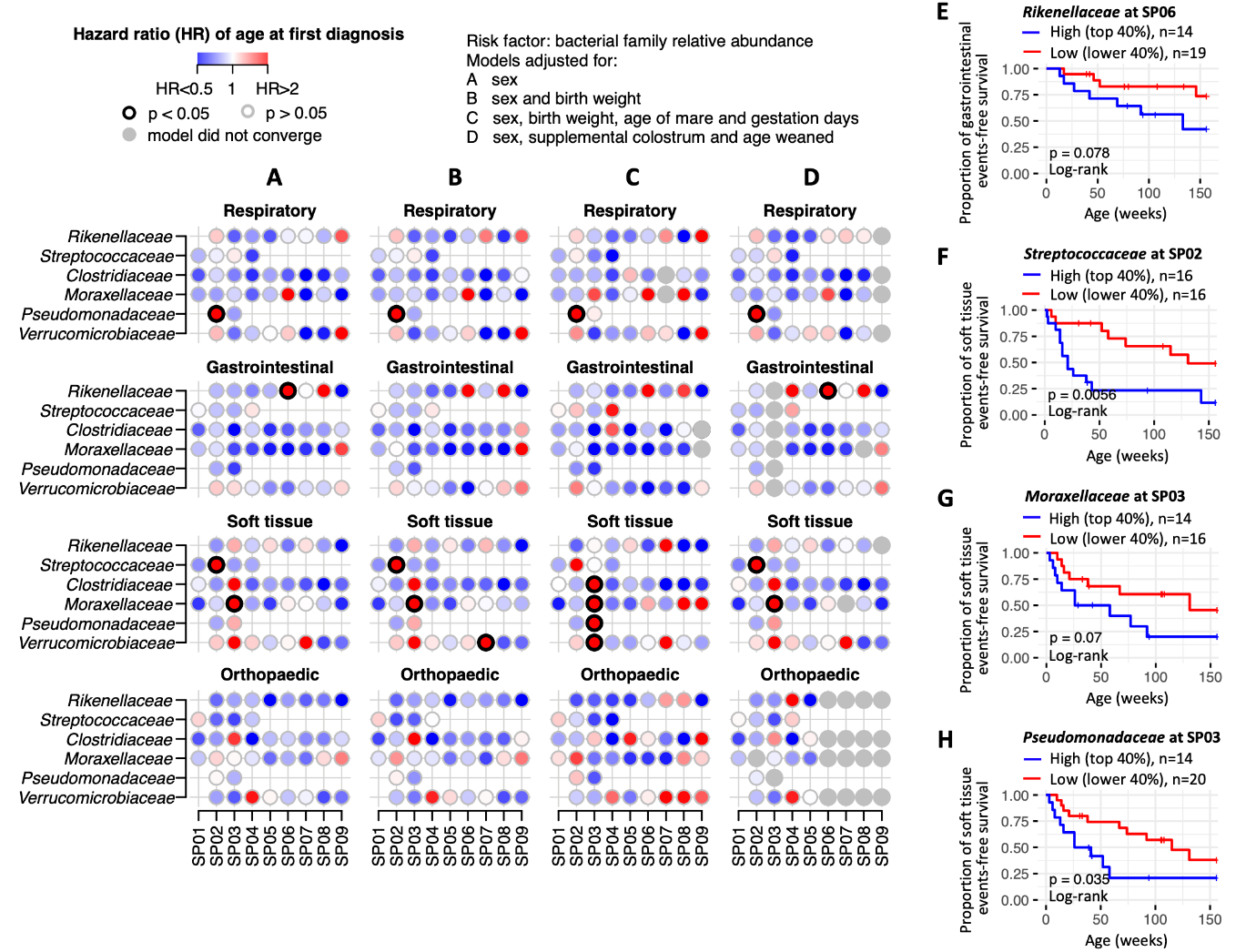


**Supplementary figure 8:** Effect of relative bacterial family abundance on age at first diagnosis. Multivariable Cox mixed-effects models were constructed per sampling point (SP01 to SP09) to assess the relationship between relative bacterial family abundance and age of first diagnosis of a health event. The models were adjusted for different sets of variables (**panels A-D**) and stud of birth was included as a random effect. A-D Dot plots show time-to-event standardised hazard ratios of respiratory, gastrointestinal, soft tissue or orthopaedic events (row sections), at each of the nine sampling points (horizontal axis), using the relative abundance of selected bacterial families as risk factors (vertical axis). Each dot corresponds to an independent model. A total of 189 bacterial families were explored but only bacterial families that have a significant hazard ratio (HR) (BH p<0.05), in at least one model, are shown. Missing dots indicate data was filtered out because of low number of counts. Dot color intensity corresponds to HR values, with HR>1 in red and HR<1 in blue shades. Dots with a black border line indicate BH p<0.05. E-H Kaplan-Meier time-to-event survival of horses stratified according to relative abundance of bacterial families as high (top 40%) and low (lower 40%) based on: **E** *Rikenellaceae* relative abundance at SP06 (90 days old) as a risk factor for gastrointestinal events, **F** *Streptococcaceae* relative abundance at SP02 (8 days old) as risk factor for soft tissue health events, **G** *Moraxellaceae* relative abundance at SP03 (14 days old) as risk factor for soft tissue health events, and **H** *Pseudomonadaceae* relative abundance at SP03 (14 days old) as risk factor for soft tissue health events.


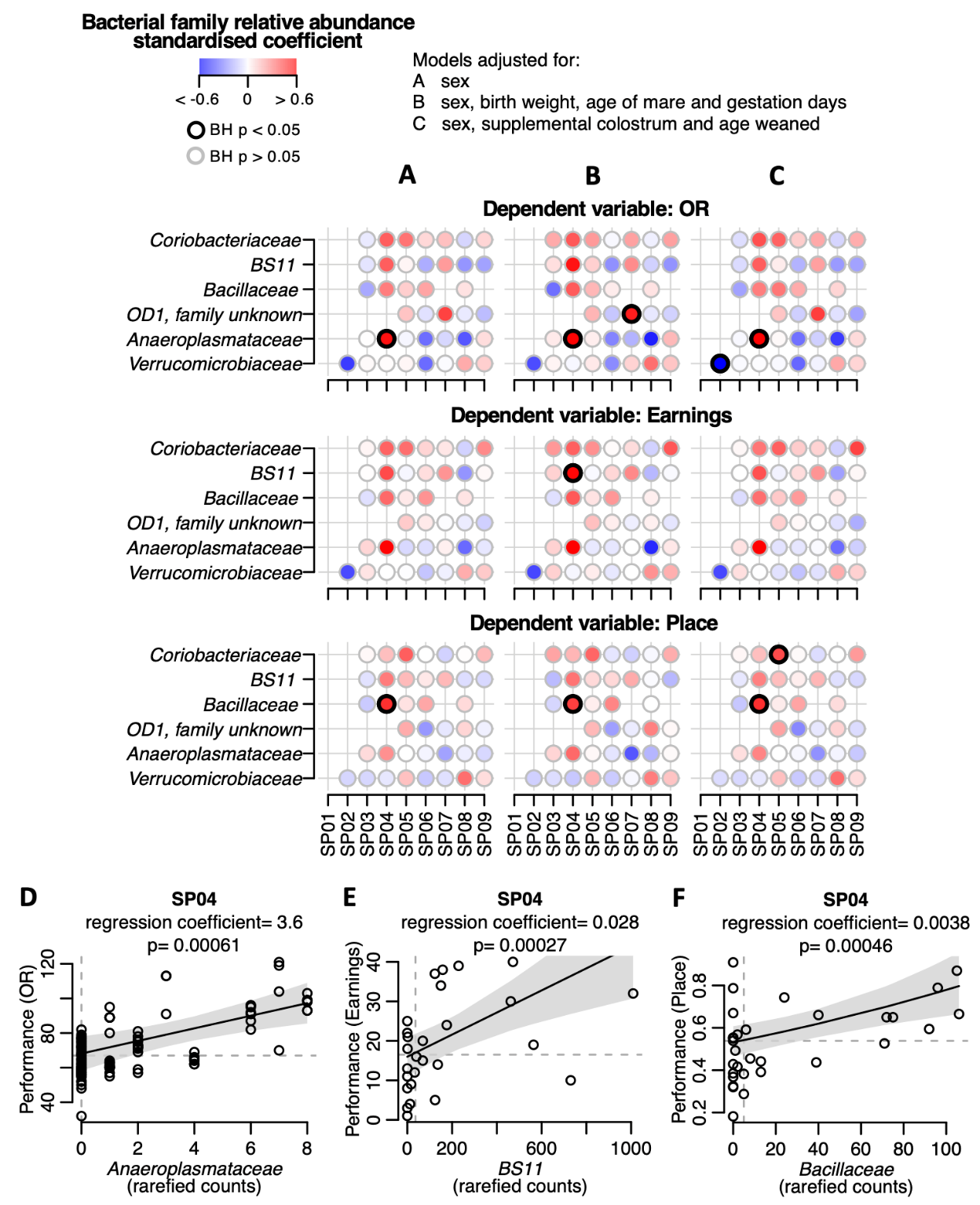


**Supplementary figure 9:** Effect of bacterial family relative abundance on racing performance. A multivariate regression model was constructed per sampling point (SP01 to SP09) to assess the relationship between bacterial family relative abundance and racing performance. The models were adjusted for different sets of variables (panels **A-C**). Official rating (OR), earnings and placing metrics were used as proxies for racing performance. **A-C** Dot plots show bacterial family relative abundance standardised regression coefficients for dependant variables OR, earnings and place. A total of 189 bacterial families were explored as predictor variables at each of the nine sampling points (horizontal axis) but only bacterial families that have a significant regression coefficient (BH p<0.05), in at least one model, are shown (vertical axis). Each dot corresponds to an independent model. Missing dots indicate data were filtered out because of low number of counts. Dot colour intensity corresponds to bacterial family relative abundance standardized regression coefficients. Dots with a black border line indicate BH p<0.05. **D-F** Performance predicted values (black line, **D** OR, **E** earnings and **F** place) and 95% confidence intervals (grey area) of the regression models when holding the covariates constant (sex for **D** and **E**; sex, birth weight, age of mare and gestation for **F**) and varying the predictor variable: **D** *Anaeroplasmataceae*, **E** *BS11*, and **F** *Bacillaceae*, at SPO4 (28 days old). Circles correspond to data points. Non-standardised regression coefficient and p-value are shown in each panel **D-F**.
